# Early childhood development when second-trimester ultrasound dating disagrees with last menstrual period: a prospective cohort study

**DOI:** 10.1186/1471-2393-12-32

**Published:** 2012-04-30

**Authors:** Jagteshwar Grewal, Meghan Wernicke, Jun Zhang

**Affiliations:** 1 Division of Epidemiology, Statistics, and Prevention Research, Eunice Kennedy Shriver National Institutes of Child Health and Human Development, 6100 Executive Boulevard, Room 7B03G, Rockville, MD, 20852, USA; 2 Division of Epidemiology, Statistics, and Prevention Research, Eunice Kennedy Shriver National Institutes of Child Health and Human Development, 6100 Executive Boulevard, Room 7B03G, Rockville, MD, 20852, USA; 3 MOE and Shanghai Key Laboratory of Children’s Environmental Health, Xinhua Hospital, Shanghai Jiao Tong University School of Medicine, 1665 Kong Jiang Road, Shanghai, 200092, P R China

**Keywords:** Gestational age discrepancy, Childhood development, Cognitive functioning, LMP

## Abstract

**Background:**

When an ultrasound-based estimate of gestational age (GA) is less (greater) than an estimate based on a definite last menstrual period, the fetus may grow slower (faster) than average. While the association between these discrepancies in GA estimates and adverse perinatal outcomes has been examined extensively, there is scant evidence about long-term effects, such as child neurodevelopment.

**Methods:**

Using data from a prospective cohort study titled, NICHD Study of Successive Small-for-Gestational Age Births, we examined if GA discrepancies in early second trimester of pregnancy (17 weeks’ gestation) are associated with: (1) impaired motor and mental function at 13 months (measured using Bayley Scales of Infant Development (Bayley)), and (2) impaired cognitive development at five years (assessed by Wechsler Preschool and Primary Scale of Intelligence – Revised Intelligence Quotient (WPPSI-R)) in the infant. The study population consisted of 572 (30% of the overall sample of 1,945) women who presented for prenatal care in Norway and Sweden between 1986 and 1988.

**Results:**

Our results showed that GA discrepancies in early second trimester are significantly associated with birthweight. We found no significant relationship, however, with the Bayley development scores at 13 months and with the WPPSI-R IQ measures at five years.

**Conclusions:**

GA discrepancies at 17 weeks’ gestation are not associated child neurodevelopment. These discrepancies do, however, relate to birthweights, providing a basis for detecting fetal growth patterns early in the second trimester of pregnancy. Our study, however, was unable to evaluate the impact of first-trimester discrepancies on impaired neurodevelopment in the infant.

## Background

Past research has shown that an ultrasound performed early in a pregnancy provides a more precise and reliable estimate of gestational age than does dating of conception based on the last menstrual period (LMP) reported by a pregnant woman [1-4]. When there is a discrepancy between ultrasound and LMP dates, the difference is typically attributed to incorrect maternal recall of LMP, as well as the higher probability of delayed rather than early ovulation [5-7]. However, the discrepancy may also indicate that the fetus is growing faster or slower than the average.

Several previous studies have collectively demonstrated an increased probability of fetal growth restriction, preterm delivery, low birth weight, stillbirth, and fetal death when the ultrasound-based gestational age estimate was lower than the LMP-based estimate [8-12]. While the relationship between these discrepancies in estimates and short-term adverse perinatal outcomes has been examined extensively, there is scant evidence about long-term effects, such as for child neurodevelopment. Moreover, the existing evaluations of the impact of restricted fetal growth on cognitive outcomes offer conflicting findings: four studies suggest that children with restricted intrauterine growth may be at a higher risk of cognitive and neurodevelopment difficulties late in life, [13-16] yet three others indicate no significant association. [17-19]

Our analysis addresses this unresolved question by using data from the *Eunice Kennedy Shriver* National Institute of Child Health and Human Development (NICHD) Study of Successive Small-for-Gestational Age Births, a prospective population-based multicenter study of women who presented for prenatal care in Norway (Trondheim and Bergen) and Sweden (Upsala) between 1986 and 1988. The objectives are to examine whether discrepancies between ultrasound- and LMP-based estimates of gestational age are associated with (1) impaired motor and mental function at 13 months, and (2) impaired cognitive development at five years in the infant.

## Methods

### Data

The data for the study were drawn from NICHD Study of Successive Small-for-Gestational Age Births, a multicenter prospective study of the etiology of intrauterine growth restriction and its consequences for child cognitive function. This research was conducted by the NICHD in collaboration with the Universities of Trondheim and Bergen in Norway and Uppsala University in Sweden. The protocol was reviewed and approved by the respective local ethical review committees for research involving human subjects, and the mothers provided written informed consent.

The study design and selection of the participants has been described elsewhere [20]. In brief, between January 1986 and March 1988, parous women at high risk for intrauterine growth restriction (IUGR) who presented for care at less than 20 weeks of gestation were recruited. Women were considered as high risk if they had any of the following risk factors: previous delivery of a low birth weight infant (below 2,750 g), previous stillbirth or neonatal death, history of two or more spontaneous abortions, history of phlebitis, initial systolic blood pressure above 140 mmHg, a previous pre-term birth, pre-pregnancy weight or weight at the first clinic visit of less than 50 kg, currently smoking or smoked at conception, and use of alcohol. In addition, to be eligible for the study the women had to be of Caucasian origin, have a singleton pregnancy, and speak one of the Scandinavian languages. All of the women received four scheduled ultrasound exams over the course of their pregnancy. Childhood motor, mental, and cognitive functioning was assessed in all infants at both 13 months and five years of age.

Of the 6,354 women referred to the study, 5,722 were eligible for participation and made an appointment. Of these women, 1,384 were classified as high risk for IUGR and enrolled for the study. In addition, a random sample of 561 women from a Nordic population of Caucasian women was selected as a reference group. Of the overall sample of 1,945 women selected to be followed closely during pregnancy, 393 were ultimately labeled as ‘drop outs’ because they failed to complete more than one of the four scheduled ultrasound examinations. No significant differences were found between women who dropped out and those who completed scheduled visits with respect to all available socio-demographic and medical variables [20]. For purposes of our analysis, we used data only from the participants who had information collected at the 13 month and/or the five-year follow-up assessment, yielding a study sample of 572 women.

At the 13-month follow-up visit, mental and psychomotor development was assessed for 495 infants using the Bayley Scales of Infant Development [21]. In particular, we used Mental Development Index (MDI) and Psychomotor Development Index (PDI) scale scores, which are nonlinear transformations of the raw scores designed to eliminate age as a variable and subject to ceiling and floor effects [21,22]. In addition, the psychometric intelligence of 483 children was assessed at five years of age using the Wechsler Preschool and Primary Scale of Intelligence – Revised Intelligence Quotient (WPPSI-R IQ) test, which reports a Full Scale Score IQ, a Performance Score IQ and a Verbal Score IQ [23].

LMP-based gestational age (LMP GA) estimates were calculated using the first menstrual day of the last menstrual period that was reported by each woman upon enrollment into the study. Ultrasound-based estimates of gestational age (U/S GA) were calculated using the following Hadlock’s formula: GA = 10.50 + 0.197(BPD)(FL) + 0.9500(FL) + 0.7300(BPD), where the fetal biparietal diameter (BPD) and femur length (FL) were estimated at 17 weeks’ gestation [24]. The discrepancy in estimates of gestational age was calculated as the difference between the two estimates (U/S GA – LMP GA).

### Statistical analysis

Linear regression models were used to assess the association between the discrepancy in estimates of gestational age at 17 weeks’ gestation and (1) Bayley’s Mental and Motor Scale Development Indexes at 13 months of age and (2) Full Scale, Performance, and Verbal Score IQs at five years of age. All analyses were conducted using SAS version 9.1 (SAS Institute, Cary, NC), with and without adjustment for the following covariates: maternal age, maternal smoking status, maternal education level, maternal socio-economic status (SES), maternal body mass index (BMI), neonatal intensive care unit (NICU) admission, and breastfeeding at three months of age. Kindergarten status was also considered for adjustment when examining the five-year follow-up data.

## Results

Overall, the discrepancy in the estimates of gestational age was within ±3 days for 49% of the study participants. The distribution of discrepancies was skewed to the left, i.e., estimates based on LMP were more likely to be higher than those based on an early second-trimester ultrasound (data not shown).

Table 1 presents select maternal and fetal characteristics of the 572 women who were included in the analysis, differentiated with respect to the categories of discrepancies in estimates of gestational age. The mean age of those in the ≤ −11 days category was lower than the overall average in the sample (28.8 years), whereas the mean age for those in the ≥ 11 days category was higher than the overall average. Mean pre-pregnancy weight and BMI both varied significantly across the five categories, exhibiting non-linear patterns: women in the ≤ −11 days and ≥ 11 days categories weighed more prior to the current pregnancy and had a considerably higher BMI than those in the intermediate categories. Meanwhile, the fetal characteristics also exhibit variation across these categories that generally follow clear monotonic patterns. Mean birth weight increased significantly as the U/S GA – LMP GA discrepancy ranged from ≤ −11 days to ≥ 11 days. Mean gestational age declined from 42.7 weeks among women with a U/S GA – LMP GA discrepancy of ≤ −11 days to 39.0 weeks among those where the discrepancy was ≥ 11 days. Among mothers with a U/S GA – LMP GA discrepancy of ≤ −11 days, equal proportions of babies were male and female. Among those with a discrepancy of ≥ 11 days, however, a significantly higher share of babies was male.

**Table 1:** Distribution of select maternal and fetal characteristics by discrepancy in gestational age at 17 weeks’ gestation

	**Discrepancy (U/S GA – LMP GA) in gestational age [days]**						
	**≤ −11**	**−10 to −4**	**−3 to +3**	**+4 to +10**	**≥ 11**	**Overall**	*p*value*
	**[N = 34]**	**[N = 91]**	**[N = 291]**	**[N = 142]**	**[N = 14]**	**[N = 572]**	
***Maternal Characteristics***							
Mean age^†^	27.9	27.6	29.0	29.2	31.8	28.8	0.001
Mean height (cm)	165.4	164.9	166.0	166.7	166.7	166.0	0.23
Mean pre-pregnancy weight (kg)	63.7	58.6	59.5	58.8	63.0	59.5	0.03
Mean pre-pregnancy BMI (kg/m²)	23.1	21.6	21.6	21.2	22.6	21.6	0.004
Education (%)							
Less than High School	39.3	22.1	14.4	19.2	14.3	18.2	0.21
High School	42.9	50.0	50.2	50.8	57.1	50.1	
College/Trade School	14.3	19.8	26.9	23.9	14.3	24.0	
More than College	3.6	8.1	8.5	6.2	14.3	7.8	
Parity (%)							
1	70.6	70.3	70.1	71.1	57.1	70.1	0.88
2	29.4	29.7	29.9	28.9	42.9	29.9	
***Fetal Characteristics***							
Mean birth weight (gms)	3256.1	3337.1	3360.5	3459.3	3814.3	3386.2	0.05
Mean gestational age at delivery (weeks) ^‡^	42.7	40.5	39.8	39.9	39.0	40.1	<0.0001
Sex (%)							
Male	50.0	56.0	46.1	57.0	71.4	51.2	0.09
Female	50.0	44.0	54.0	43.0	28.6	48.8	

U/S GA: Ultrasound-based gestational age; LMP GA: Last Menstrual Period-based gestational age.

* *p* values based on one-way ANOVA for continuous variables and the X² test for categorical variables.

^†^ Mean age is calculated from date of reported LMP and date of birth.

^‡^ Gestational age at delivery is based on LMP.

Table 2 shows the mean MDI and PDI scale scores measured at 13 months and the Full Scale Score IQ, Performance Score IQ and Verbal Score IQ assessed at five years of age. There were no differences in any of these measures of cognitive development across the categories of discrepancies in gestational age. Different groupings of discrepancies were compared in this same manner (data not shown), again with insignificant results.

**Table 2:** Distribution of motor, mental, and cognitive development scores by discrepancy in gestational age at 17 weeks’ gestation

	**Discrepancy (U/S GA – LMP GA) in gestational age (days)**						
	**≤ −11**	**−10 to −4**	**−3 to +3**	**+4 to +10**	**≥ 11**	**Overall**	*p*value*
	N = **34**	N = **91**	N = **291**	N = **142**	N = **14**	N = **572**	
***13 month: Bayley Score***							
Mean Bayley Score: MDI	112.9	116.2	113.7	114.4	118.9	114.4	0.36
Mean Bayley Score: PDI	106.0	106.3	106.6	107.5	107.0	106.8	0.97
***5 year: WPPSI-R IQ***							
Mean Full Scale Score IQ	104.7	107.1	107.9	107.0	109.9	107.4	0.81
Mean Performance Score IQ	105.6	108.6	110.0	109.0	114.0	109.4	0.46
Mean Verbal Score IQ	102.9	103.9	104.1	103.2	104.1	103.7	0.98

U/S GA: Ultrasound-based gestational age; LMP GA: Last Menstrual Period-based gestational age; MDI: Mental Development Index; PDI: Psychomotor Development Index.

* *p* values based on one-way ANOVA.

Table 3 presents the adjusted results of linear regression models used to assess the association between the discrepancy in estimates of gestational age at 17 weeks’ gestation and motor and mental function at 13 months, as well as the cognitive functioning at five years of age. No significant relationships were observed between the gestational age discrepancies and any of the measures of early childhood motor, mental, and cognitive development. Adjustment of the risk estimates for maternal age, maternal smoking status, education level, SES, BMI, breastfeeding at three months of age, NICU admission, sex, and kindergarten status (five-year follow-up only) did not yield results that were sufficiently different to change the interpretation associated with the crude risk estimates (data not shown).

**Table 3:** Adjusted* association between discrepancy in gestational age at 17 weeks’ gestation and childhood motor, mental, and cognitive development

	**β**^†^	**S.E.**	***p *value**
***13 month: Bayley Score***			
Bayley Score: MDI	0.03	0.10	0.79
Bayley Score: PDI	0.05	0.12	0.70
***5 year: WPPSI-R IQ***			
Full Scale Score IQ	0.05	0.12	0.69
Performance Score IQ	0.00	0.10	1.00
Verbal Score IQ	−0.02	0.11	0.82

MDI: Mental Development Index; PDI: Psychomotor Development Index.

* Adjusted for maternal age, maternal smoking status, maternal education level, maternal SES, maternal BMI, breastfeeding at 3 months of age, NICU admission, sex of baby, and kindergarten status (adjusted only for the 5-year follow up data).

^†^ The β coefficients represent mean difference in the Bayley and the WPPSI-R IQ scores per unit change in gestational age discrepancy (days).

## Discussion

Our large prospective study showed that discrepancies between early second trimester ultrasound-based and LMP-based estimates of gestational age are significantly associated with birthweight, indicating that fetal growth patterns (slow or fast) are already evident in early 2nd trimester of pregnancy. We found no significant relationship, however, between gestational age discrepancies at 17 weeks’ gestation and the Bayley mental and psychomotor development scores measured at 13 months. Likewise, there was no association between the gestational age discrepancies and the WPPSI-R full scale, performance, and verbal IQ measures assessed at five years of age.

Discrepancies in gestational age estimates, i.e., where the LMP-based estimates exceed those estimated by an ultrasound, have been associated with increased risk of birth weight less than 2500 g and of small for gestational age infants. [8,25] Moreover, these differences in estimates may also indicate the presence of early fetal growth restriction, as demonstrated by Morin, *et al.* (2005) in a hospital-based cohort study of 46,514 Canadian women [12]. The authors concluded that even though LMP-based estimates of gestational age often exceed those based on an ultrasound due to errors in menstrual dating, the discrepancy may also be associated with early fetal growth restriction.

To date, the studies that have specifically examined the relationship between restricted intrauterine growth and impaired cognitive outcome offer conflicting findings. For example, Fattal-Valevski, *et al.* (1999) compared 85 infants with intrauterine growth retardation with 42 control infants and concluded that at three years of age, neurodevelopmental scores for children with growth restrictions were significantly lower than those for the controls [14]. These results were substantiated by two subsequent studies, [15,16] but contradicted by three others [17-19]. Our findings of no significant impact of intrauterine growth restriction on a variety of neurodevelopmental outcomes in children are consistent with the latter set of results.

At least two reasons may account for these findings of no association in this prior research as well as our own study. To begin with, the discrepancy between the ultrasound- and LMP-based estimates of gestational age may be due to several factors, including erroneous LMP, physiologically small (or large) fetus, and pathological growth restriction (or overgrowth). Yet erroneous LMP cannot completely explain the significant increase in mean birthweight increased observed as the U/S GA – LMP GA discrepancy ranged from ≤ −11 days to ≥11 days. Meanwhile, the physiological difference in fetal size can be controlled, to some extent, by adjusting for maternal BMI, parity and the sex of the baby. By implication, pathological fetal growth likely affects the discrepancy in the gestational age estimates, albeit the extent of its contribution is unknown. Also, it may be that any impact of pathological fetal growth on gestational age discrepancies is not substantial enough to yield statistically significant difference in child neurodevelopment. A limitation of our study is that we lacked information on Doppler flow and thus could not distinguish between infants who were pathologically growth restricted as versus constitutionally small.

Another plausible explanation may lie in the role of postnatal “catch-up” growth among small-for-gestational-age infants. There is evidence that postnatal growth patterns, rather than appropriateness of weight for gestational age, is actually the key determinant of neurodevelopmental outcomes later in life [26,27]. For example, Latal-Hajnal, *et al.* (2003) demonstrated that small-for-gestational-age infants who showed substantial catch-up growth, with weight above the 10th percentile at two years of age, had neurodevelopmental outcomes comparable to appropriate-for-gestational-age children whose weight remained in the appropriate range at the two year age milestone [27]. In the context of our study, therefore, postnatal catch-up growth among the growth restricted fetuses may have compensated for the effects of pathological fetal growth on child neurodevelopment that we might otherwise have expected to observe.

A further important consideration is the methodological differences among the prior studies, which also diminish their comparability to the current study. For example, Leonard, *et al.* (2007) [16] and Hutton, *et al.* (2007) [17] used the ratio of the observed birthweight to the expected birthweight for a given gestational age to assess fetal growth, whereas Leitner, *et al.* (2007) [15] and Gortner, *et al.* (2003) [19] classified all infants with birthweights below the 10^th^ percentile as small for gestational age at birth. All of these studies examine the impact of low birth weight alone on the risk of poor neurodevelopmental outcomes. None of the studies take into account when the in utero failure in growth commenced.

The impact of differing in utero growth patterns on neurovelopment was demonstrated by Harvey, *et al.* (1982). [28] This study monitored the intrauterine growth of 51 small-for-gestational-age infants using serial ultrasound cephalometry and concluded that children whose head growth began to slow down before 26 weeks’ gestation had significantly lower developmental scores relative to those whose head growth slowed down later in pregnancy. Likewise, our hypothesis was that poor intrauterine growth in the early second trimester, as gauged by the discrepancy in ultrasound- and LMP-based estimates of gestational age, would be reflected in poor cognitive development in the infants at both 13 months and five years of age. We find, however, no such associations.

Meanwhile, most of the relevant literature concerning the relationship between intrauterine growth and cognitive development has focused on the impact of growth restriction. Far less research has been devoted to examining whether there is any link between large babies and poor development outcomes. A rare study in this vein was Ounsted, *et al.* (1983) [29], which compared 212 large-for-dates infants with 236 controls. They concluded that development scores at four years of age were marginally higher in the former group as compared to in the latter group; however, there was so significant difference between the two groups after adjustment for sex and social class. Similarly, our study found no association between large-for-dates infants and cognitive development at either 13 months or five years of age.

Valid concerns exist about generalizing the findings of this study, based on analysis using data collected nearly 25 years ago, to the current obstetric population and clinical practices worldwide. In the interim, the value of a first-trimester dating ultrasound, now performed in several countries, has been demonstrated. For example, a recent study showed an increased risk of adverse birth outcomes (e.g., preterm birth, low birth weight, small for gestational age at birth) associated with fetal growth restriction during the first trimester (i.e., from 10 weeks 0 days to 13 weeks 6 days) [30]. Unfortunately, we lack information from a first-trimester ultrasound; in our data, the initial ultrasound was perfomed at 17 weeks gestation. Consequently, we cannot evaluate the impact of gestational age discrepancies during the first trimester on either birth weight or subsequent cognitive development in the infant. Examining whether first trimester discrepancies are associated with child neurodevelopment would definitely be worthwhile, given the obvious relevance for clinical practice. Yet we can surmise from the results of this study that any such relationship is unlikely, given that no association was found between discrepancies during the second trimester, which tend to be greater than those generally observed during the first trimester, and differences in cognitive development in the infant.

## Conclusion

In conclusion, our prospective cohort study of low-risk, pregnant women demonstrated that gestational age discrepancies at 17 weeks’ gestation are not associated with poor cognitive development in the infants at both 13 months and five years of age. These gestational age discrepancies do, however, relate to birthweights, thereby providing a basis for detecting fetal growth patterns early in the second trimester of pregnancy.

## Competing interest

The authors declare that they have no competing interest.

## Authors’ contributions

JG and JZ were responsible for the study concept and design. MW conducted the statistical analysis. JG, MW, and JZ participated in the interpretation of the data and in the drafting of the manuscript. JG provided critical revisions of the manuscript and JZ contributed important intellectual content. All authors read and approved the final manuscript.

## Funding

This research was supported by the Intramural Research Program of the NIH, *Eunice Kennedy Shriver* National Institute of Child Health and Human Development.

## Pre-publication history

The pre-publication history for this paper can be accessed here:

http://www.biomedcentral.com/1471-2393/12/32/prepub
